# Twist1-mediated 4E-BP1 regulation through mTOR in non-small cell lung cancer

**DOI:** 10.18632/oncotarget.5026

**Published:** 2015-08-12

**Authors:** Tangfeng Lv, Qian Wang, Meghan Cromie, Hongbing Liu, Song Tang, Yong Song, Weimin Gao

**Affiliations:** ^1^ Department of Environmental Toxicology, The Institute of Environmental and Human Health, Texas Tech University, Lubbock, Texas 79416, United States of America; ^2^ Department of Respiratory Medicine, Jinling Hospital, Nanjing, Jiangsu 210002, China; ^3^ Department of Respiratory Medicine, Jiangsu Province Hospital of Chinese Medicine, Affiliated Hospital of Nanjing University of Chinese Medicine, Nanjing, Jiangsu 210029, China

**Keywords:** Twist1, epithelial-mesenchymal transition, phosphorylated 4E-binding protein 1, phosphorylated mammalian target of rapamycin, non-small cell lung cancer

## Abstract

Twist1 overexpression corresponds with poor survival in non-small cell lung cancer (NSCLC), but the underlining mechanism is not clear. The objective of the present study was to investigate the tumorigenic role of Twist1 and its related molecular mechanisms in NSCLC. Twist1 was overexpressed in 34.7% of NSCLC patients. The survival rate was significantly lower in patients with high Twist1 expression than low expression (*P* < 0.05). Twist1 expression levels were higher in H1650 cells, but relatively lower in H1975 cells. H1650 with stable Twist1 knockdown, H1650shTw, demonstrated a significantly slower rate of wound closure; however, H1975 with stable Twist1 overexpression, H1975Over, had an increased motility velocity. A significant decrease in colony number and size was observed in H1650shTw, but a significant increase in colony number was found in H1975Over (*P* < 0.05). Tumor growth significantly decreased in mice implanted with H1650shTw compared to H1650 (*P* < 0.05). 4E-BP1 and p53 gene expressions were increased, but p-4E-BP1 and p-mTOR protein expressions were decreased in H1650shTw. However, 4E-BP1 gene expression was decreased, while p-4E-BP1 and p-mTOR protein expressions were increased in H1975Over. p-4E-BP1 was overexpressed in 24.0% of NSCLC patients. Survival rate was significantly lower in patients with high p-4E-BP1 expression than low p-4E-BP1 (*P* < 0.01). A significant correlation was found between Twist1 and p-4E-BP1 (*P* < 0.01). A total of 13 genes in RT-PCR array showed significant changes in H1650shTw. Altogether, Twist1 is correlated with p-4E-BP1 in predicting the prognostic outcome of NSCLC. Inhibition of Twist1 decreases p-4E-BP1 expression possibly through downregulating p-mTOR and increasing p53 expression in NSCLC.

## INTRODUCTION

Approximately 85% of lung cancer cases are classified as non-small cell lung cancer (NSCLC), which is often associated with a serious prognosis [1]. Contributing to a poor prognosis is the ability of cancer cells to metastasize [2]. Metastasis is characterized as the extravasation of cancer cells through the circulation and further colonization into new target sites [3, 4]. Epithelial-mesenchymal transition (EMT), a well-known process involving the loss of cell-cell adhesion in epithelial cells and promoting a migratory and invasive phenotype [5], plays a critical role in cancer metastasis [1] and is even believed to be an initial step of metastasis [6]. A notable hallmark of EMT is the loss of E-cadherin-mediated cell-cell adhesion and the subsequent gain of N-cadherin expression, which is tightly regulated by Twist1, a basic helix-loop-helix (bHLH) transcription factor, and other transcription factors [7, 8].

Twist1 overexpression has been frequently found and documented in different types of cancer, including breast cancer [9, 10], gastric cancer [11, 12], hepatocellular carcinoma [13], NSCLC [14–17], prostate cancer [18, 19], and sarcomas [20], and it is becoming an important diagnostic and prognostic marker for cancer detection and monitoring in patients. Twist1 has been shown to inhibit apoptosis and/or promote cell survival in cells that have been activated by oncogenes [21, 22]. The upstream or downstream signaling pathways of Twist1 are not completely understood, although there is supportive information that Twist1 is upregulated by classical EMT-inducing pathways during development, inflammation, and cancer [6, 23]. For instance, the connections between Twist1 and phosphoinositide 3-kinase (PI3K)/protein kinase B (PKB/Akt)/mammalian target of rapamycin (mTOR), Ras/ERK, signal transducer and activator of transcription-3, p53, Wnt, histone acetyltransferase 1-interacting factor 1, and NF-κB signaling pathways has been reported [24, 25]. As an example, the suppression of mTOR activity and the decrease of mTOR phosphorylation have been observed in lung cancer H1299 cells after Twist1 knockdown [17]. mTOR could act as an important regulator for 4E-BP1 phosphorylation, and p-4E-BP1 has been frequently found and/or indicated as a prognostic predictor for poor survival in different cancers such as breast cancer [26], nasopharyngeal carcinoma [27], hilar cholangiocarcinoma [28], gastric cancer [29], and lung cancer [30, 31].

Studies have shown the clinical significance of Twist1 in NSCLC [14–17, 32, 33], suggesting a reduced survival in patients with Twist1 overexpression; however, the specific molecular mechanisms driven by Twist1 in NSCLC are not clearly understood. In the present study, the role of Twist1 and its molecular mechanisms were investigated in NSCLC. *In vivo* and *in vitro* studies were implemented to gain a comprehensive analysis of Twist1 in human patients, animal models, and human NSCLC cell lines. Twist1 overexpression was frequently found in tumor tissues from NSCLC patients and associated with a significantly lower survival rate. The overexpression of Twist1 was found to play a significant role in tumorigenicity, as reflected by slower rates of wound closure and a decrease in colony number in H1650shTw cells, but with converse findings in H1975Over cells. The xenograft mouse model further indicated that mice injected with H1650shTw cells revealed smaller tumors compared to H1650-induced tumors. Additionally, 4E-BP1 and p53 were upregulated, while p-4E-BP1 and p-mTOR were decreased in H1650shTw cells. On the other hand, 4E-BP1 was decreased, while p-4E-BP1 and p-mTOR were increased in H1975Over cells. Finally, the overexpression of p-4E-BP1 was associated with poor survival in NSCLC patients and significantly correlated with Twist1 overexpression. Overall, these findings provide additional evidence of the clinical importance of Twist1 in NSCLC, possibly through the regulation of 4E-BP1.

## RESULTS

### Twist1 expression in human lung cancer and paracancerous tissues and its association with clinicopathological parameters

**Figure 1:**
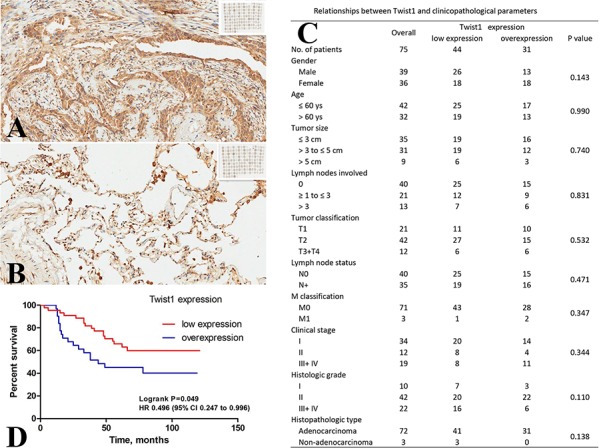
Clinical significance of Twist1 in NSCLC Assessment of Twist1 expression by IHC **A & B.** Representative microphotographs (20 ×) of Twist1 expression in lung cancer tissues (A) and paracancerous tissues (B). Twist1 positive staining was seen in cytoplasm and nucleus. High expression levels of Twist1 were found in lung tumors (A), whereas the expression was low in lung cancer paracancerous tissues (B). Relationships between Twist1 and clinicopathological parameters in 75 cases of NSCLC patients **C.** Kaplan-Meier survival curves of NSCLC patients stratified according to Twist1 expression (log-rank test, *P* < 0.05)**D**.

We examined the difference in protein expression of Twist1 between human lung cancer and paracancerous tissues from TMA (Fig. 1A & 1B). Twist1 was mainly found in the nucleus and cytoplasm of cells and was restricted to tumor glands in IHC staining. Twist1 overexpression was observed in 34.7% (26 of 75) of lung cancer tissues tested, but not in the paracancerous tissues (*P* < 0.01). The relationships between Twist1 expression and clinicopathological parameters are shown in Figure 1C. No significant associations were observed between Twist1 expression and gender, age, tumor size, lymph nodes involved, tumor classification, lymph node status, metastasis (M) classification, clinical stage, histologic grade, and histopathologic type. Kaplan-Meier survival curves were further constructed to evaluate whether the expression of Twist1 in primary lung cancer was associated with the patient's outcome. A significant correlation between the immunointensity of Twist1 and the survival of patients was shown (log-rank test, *P* < 0.05). Survival was significantly lower in patients with Twist1 overexpression than those with low Twist1 expression (*P* = 0.049, Fig. 1D).

### Twist1 expression in lung cancer cell lines, Twist1 shRNA knockdown in H1650 cells, and Twist1 overexpression in H1975 cells

**Figure 2:**
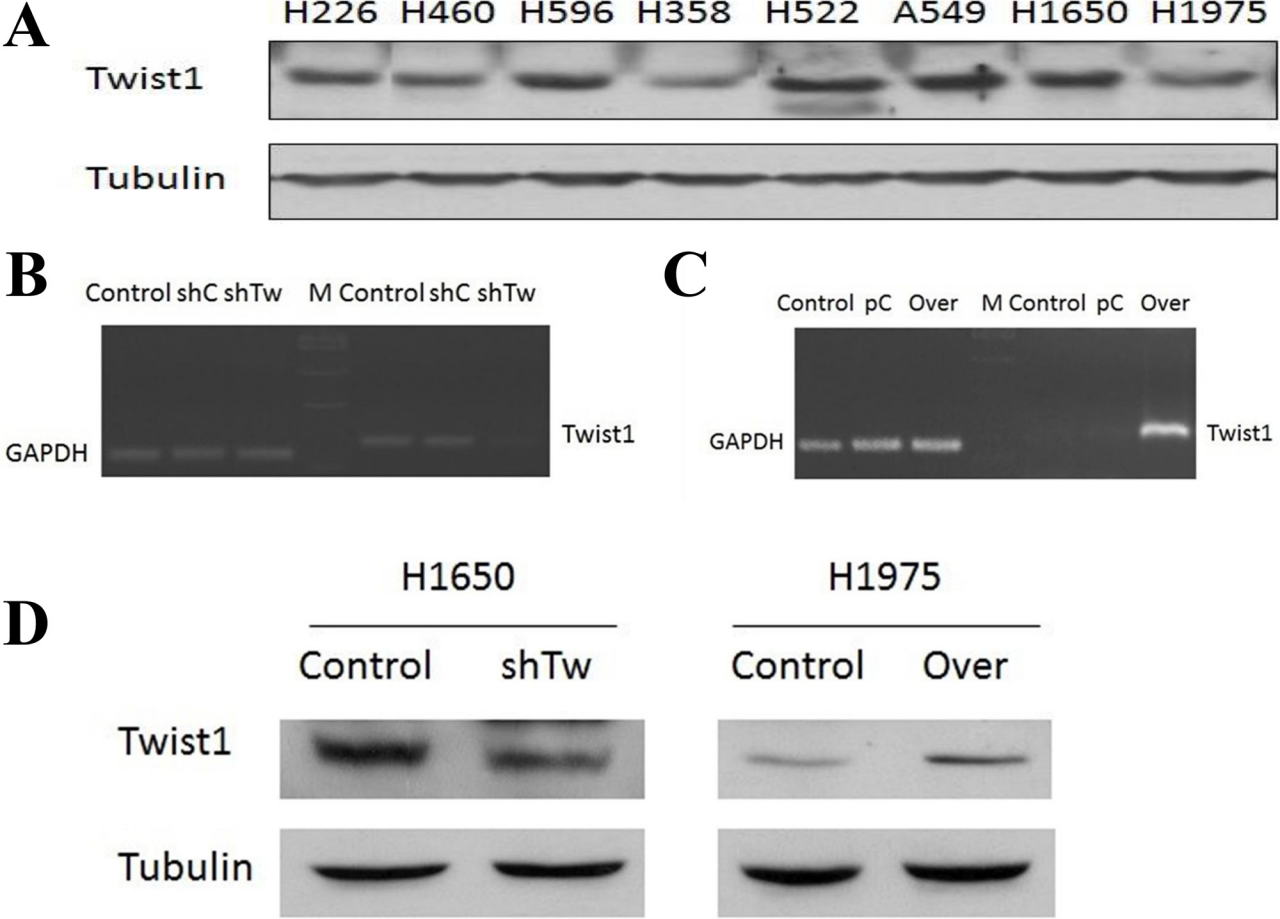
Twist1 expression in NSCLC cell lines and Twist1 modulation in H1650 and H1975 Relative expression levels of Twist1 protein were analyzed in 8 different lung cancer cell lines by Western blot **A.** Cells with stable Twist1 knockdown conducted in H1650 cells, named as H1650shTw, while cells with stable Twist1 overexpression conducted in H1975 cells, named as H1975Over. The gene expression of Twist1 in H1650 and H1650shTw **B.** and in H1975 and H1975Over **C.** The protein expression of Twist1 in H1650 and H1650shTw and in H1975 and H1975Over **D.**

The relative expression level of Twist1 was analyzed by Western blot in a panel of 8 lung cancer cell lines, among which A549, H460, and H1650 are p53 wild type, H522, H596, and H1975 are p53 mutant, and H358 is p53 null. Twist1 expression levels were higher in A549, H596, and H1650 cell lines, but relatively lower in H1975 and H358 cell lines (Fig. 2A). To explore the role of Twist1 in human NSCLC, we employed shRNA to silence Twist1 in H1650 cells. We have demonstrated that shTw significantly suppressed Twist1 at both the transcript and protein levels in H1650 cells (Fig. 2B & 2D). The mRNA expression of Twist1 decreased by 4.5 fold in H1650shTw cells compared to H1650 cells or H1650 cells transfected with the control plasmid (Fig. 2B). On the other hand, Twist1 expression levels of both mRNA and protein were significantly increased in H1975 cells transfected with Over (Fig. 2C & 2D). There was more than a 1,000 fold increase of Twist1 mRNA expression in H1975Over compared to H1975 cells or H1975 cells transfected with the control plasmid (Fig. 2C).

### Migration assay, soft agar assay, and the nude mouse xenograft model

**Figure 3:**
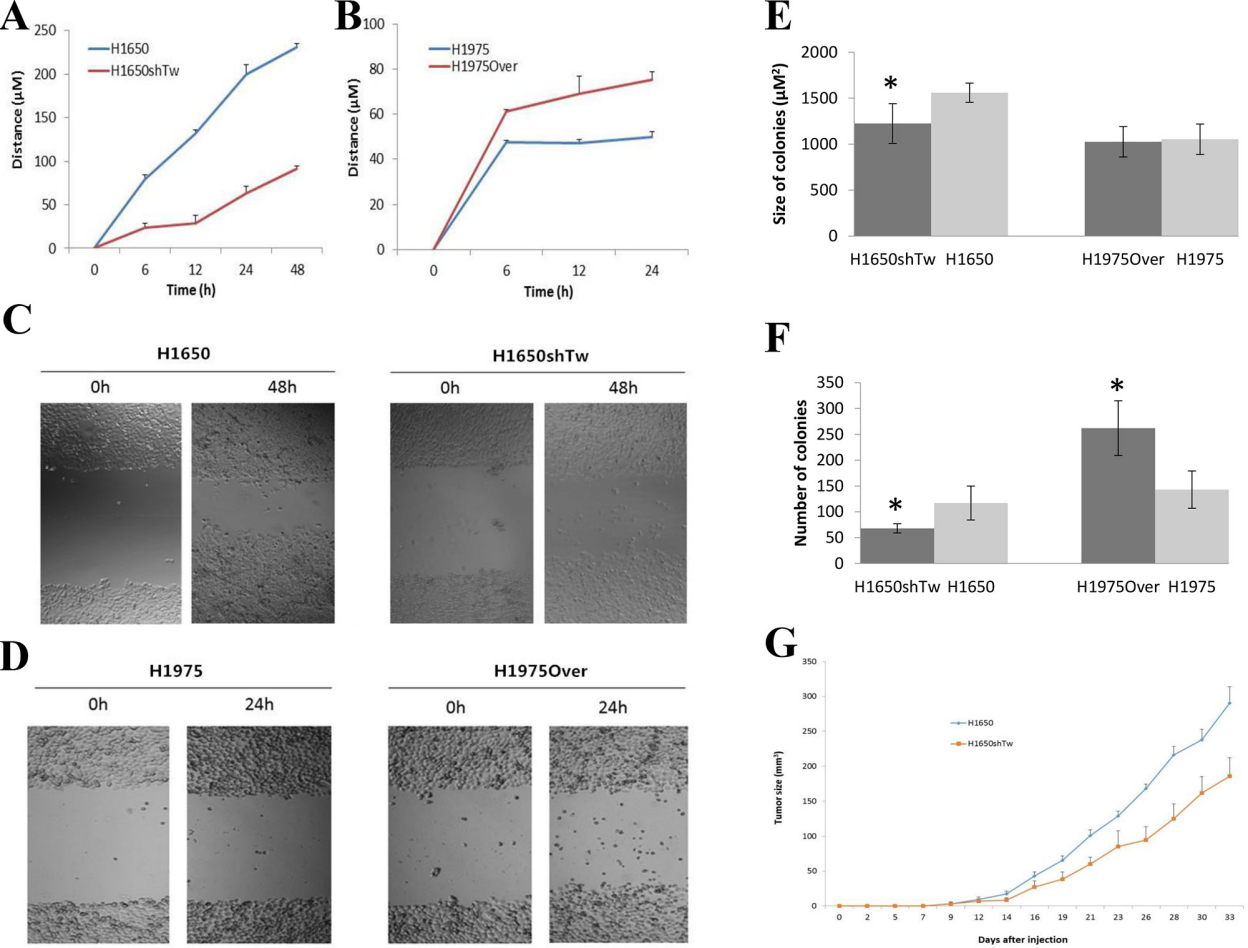
Effects of Twist1 on scratch and colony assays and *in vivo* xenograft tumor growth Scratch assay in H1650 **A.** and H1975 **B.** after Twist1 modulation. Cells were allowed to migrate in control conditions or in the presence of Twist1 knockdown or overexpression. Images were obtained at 0 and 48 h in H1650 **C.** and at 0 and 24 h in H1975 **D.** after scratch formation. The effects of Twist1 on size **E.** and the number of colonies **F.** H1650shTw had a significantly smaller colony number and colony size compared to H1650 (*P* < 0.05), and H1975Over had an increased number of colonies (*P* < 0.05), but a similar size of colonies compared to H1975. *In vivo* xenograft tumor growth after implantation of H1650 and H1650shTw in nude mice **G.** Knockdown of Twist1 inhibits tumor growth (measured by tumor size) in mice implanted with H1650shTw compared to those with H1650.

The role of Twist1 on the motility of H1650 and H1975 cells was examined using the wound healing assay. As shown in Fig. 3A–3D, H1650shTw cells demonstrated a significantly slower rate of wound closure compared to H1650 cells at 6, 12, 24, and 48 h (*P* < 0.05); however, H1975Over cells had an increased motility velocity as compared to H1975 cells at 6, 12, and 24 h (*P* < 0.05). These results suggest that Twist1 plays a critical role in cell motility for lung cancer cells.

To determine the tumorigenic potential of Twist1 in lung cancer cells, soft agar assay was implemented. A significant decrease in colony number and size was observed in H1650shTw cells compared to H1650 cells (*P* < 0.05, Colony size: 1,224 ± 216.2 in H1650shTw & 1,560 ± 105.3 μM^2^ in H1650; Colony number: 68 ± 9 in H1650shTw & 117 ± 33 in H1650, Fig. 3E & 3F). However, a significant increase in colony number, but not colony size, was found in H1975Over cells compared to H1975 cells (*P* < 0.05, Colony size: 1,026 ± 165.7 in H1975Over & 1,054 ± 165.6 μM^2^ in H1975; Colony number: 262 ± 53 in H1975Over & 143 ± 36 in H1975, Fig. 3E & 3F). These data suggest that Twist1 plays a critical role in lung tumorigenesis.

To confirm whether Twist1 controls tumor growth *in vivo*, we generated xenograft tumors by subcutaneous injection of H1650shTw or H1650 cells. We found that tumor growth significantly decreased in mice implanted with H1650shTw cells compared to those implanted with H1650 cells (*P* < 0.05, Fig. 3G). These results indicate that silencing Twist1 inhibits xenografted tumor growth in mice.

### Twist1-mediated 4E-BP1 and mTOR regulation, and the relationship between p-4E-BP1 expression and clinicopathological parameters and Twist1 in human lung cancer

**Figure 4:**
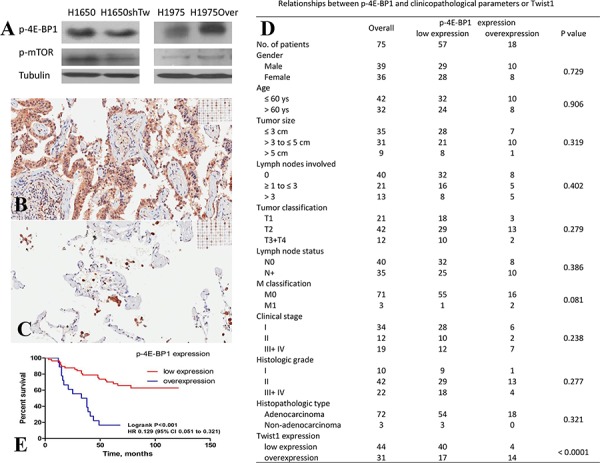
Effects of Twist1 on 4E-BP1 and mTOR, and the clinical significance of p-4E-BP1 in NSCLC The protein expressions of p-4E-BP1 and p-mTOR were decreased in H1650shTw compared to H1650, but increased in H1975Over compared to H1975 **A.** Assessment of p-4E-BP1 expression by IHC **B** & **C.** Representative microphotographs (20 ×) of p-4E-BP1 expression in lung cancer tissues (B) and paracancerous tissues (C) p-4E-BP1 positive staining was seen in cytoplasm and nucleus. High expression levels of p-4E-BP1 were found in lung tumors, whereas the expression was low in lung cancer paracancerous tissues. Relationships between p-4E-BP1 and clinicopathological parameters in 75 cases of NSCLC patients **D.** Kaplan-Meier survival curves of NSCLC patients stratified according to p-4E-BP1 expression (log-rank test, *P* < 0.05)**E**.

The differential protein expressions of Akt, p-Akt (Ser473/Thr308), eIF4E, p-eIF4E (Ser209), p-4E-BP1 (Ser65), eIF2α, and p-eIF2α (Ser51) in H1650shTw and H1650 cells were screened by multi-blot (data not shown). The expression of p-4E-BP1 was significantly downregulated in H1650shTw cells compared to H1650 cells, and this data was further confirmed by Western blot (a 2.8 fold decrease, Fig. 4A). Additionally, qRT-PCR analysis showed a 1.6 fold increase of 4E-BP1 mRNA expression in H1650shTw cells compared to H1650 cells (*P* < 0.05). Furthermore, RT-PCR showed a 1.3 fold decrease of 4E-BP1 in H1975Over compared to H1975, and Western blot revealed a significant increase (1.6 fold) of p-4E-BP1 in H1975Over compared to H1975. p-mTOR (Ser2448) was further analyzed by Western blot, and it showed a 2.0 fold decrease in H1650shTw compared to H1650 and a 1.8 fold increase in H1975Over compared to H1975 (Fig. 4A). Additionally, the gene expression of mTOR was not significantly changed in H1650shTw compared to H1650, and in H1975Over compared to H1975.

Based on these findings, IHC analysis of p-4E-BP1 was conducted in the same TMA as used for Twist1 IHC. Similar to Twist1 staining, p-4E-BP1 was localized in the nucleus and cytoplasm of cells, but was restricted to tumor glands, as observed by IHC staining (Fig. 4B & 4C). p-4E-BP1 overexpression was observed in 24.0% (18 of 75) of lung cancer tissues tested, but not in the paracancerous tissues (*P* < 0.01). The relationships between p-4E-BP1 expression and clinicopathological parameters are shown in Fig. 4D. No significant associations were observed between p-4E-BP1 expression and gender, age, tumor size, lymph nodes involved, tumor classification, lymph node status, M classification, clinical stage, histologic grade, and histopathologic type. Kaplan-Meier survival curves were further constructed to evaluate whether the expression of p-4E-BP1 was associated with the patient's outcome. The survival rate was lower in patients with p-4E-BP1 overexpression than those with low p-4E-BP1 expression (*P* < 0.01, Fig. 4E).

Finally, we evaluated the association between Twist1 and p-4E-BP1 protein expressions. A significant correlation was found between Twist1 and p-4E-BP1 by Spearman rank correlation test (*P* < 0.01, Fig. 4D). Moreover, IHC scoring of Twist1 and p-4E-BP1 was used to categorize the co-overexpression of Twist1 and p-4E-BP1 among the 75 lung cancer patients tested for IHC. The association between Twist1/p-4E-BP1 co-overexpression and clinicopathological parameters was also assessed. Similar to those observed in Twist1 and p-4E-BP1, no significant difference was observed in Twist1/p-4E-BP1 co-overexpression and clinicopathological parameters (data not shown). The survival rate was lower in patients with Twist1/p-4E-BP1 co-overexpression than those with low Twist1/p-4E-BP1 expression or with either Twist1 or p-4E-BP1 overexpression (*P* < 0.01, data not shown).

### RT-PCR array analysis and qRT-PCR in H1650shTw compared to H1650 cells

**Figure 5:**
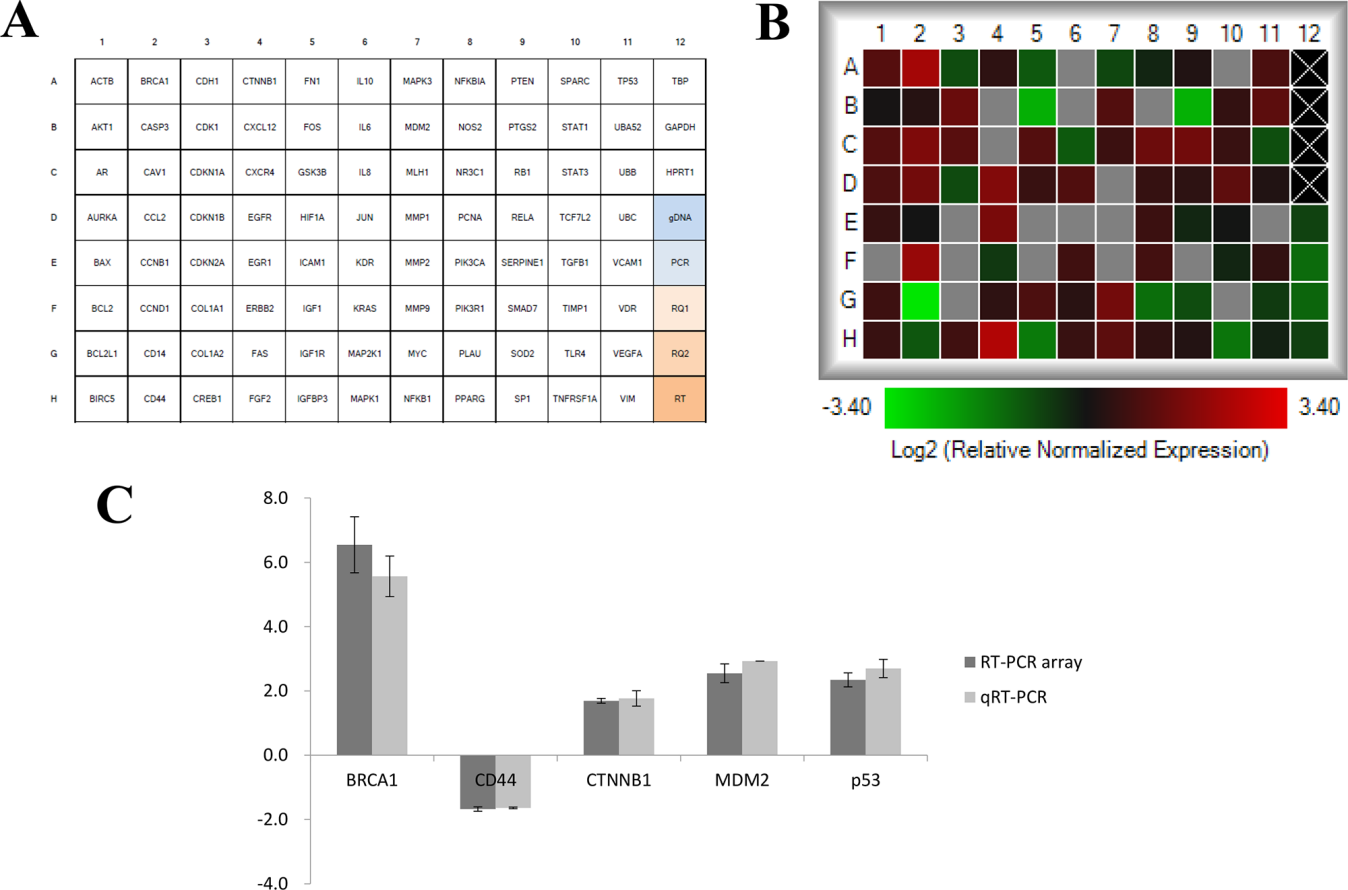
RT-PCR and qRT-PCR in H1650shTw and H1650 cells A total of 88 genes of interest were compared between H1650shTw to H1650 using RT-PCR array **A.** The heat map showed fold changes of gene expression data in H1650shTw compared to H1650. Undetected genes are labeled with gray **B.** Gene expression data of BRCA1, CD44, CTNNB1, MDM2, and p53 from both RT-PCR array and qRT-PCR **C.**

RT-PCR array was performed to profile the differential gene expression between H1650shTw and H1650 cells. Duplicate samples from each group generated reproducible results, with an average of 1.28% (range: 0.03–4.53%) for the coefficient of variation of the duplicate samples for all the measurements in the array. Among the 88 genes analyzed, a total of 19 genes were not detected. From the 69 detectable genes, 13 genes showed significant changes in H1650shTw cells compared to H1650 cells (*P* < 0.05, Fig. 5, Increased: ACTB, BRCA1, CAV1, CCND1, CDK1, GSK3B, MYC, NR3C1, and TCF7L2; Decreased: CD14, FOS, IGFBP3, and PTGS2). Verification data of 5 randomly selected genes (BRCA1, CD44, CTNNB1, MDM2, and p53) using qRT-PCR with our designed primers showed similar results as reported by RT-PCR array.

## DISCUSSION

In the present study, we demonstrated the following: (1) Twist1 overexpression is significantly associated with reduced survival of NSCLC patients; (2) knockdown of Twist1 suppresses cell invasion, clone formation, and *in vivo* tumor growth, but overexpression of Twist1 enhances cell invasion and clone formation; (3) knockdown of Twist1 suppresses p-4E-BP1 possibly through p-mTOR inhibition, and Twist1 expression is correlated with p-4E-BP1 expression in NSCLC patients; and (4) a variety of gene expressions are changed after Twist1 knockdown. For instance, FOS and PTGS2 are decreased while BRCA1 and p53 are increased after Twist1 knockdown.

Twist1 plays an important role in embryonic development and metastasis [20, 24]. Twist1 overexpression has also been found in a variety of tumors, including NSCLC [14–17]. In this study, we examined the expression of Twist1 in lung tissues from 75 NSCLC patients. We found that the overexpression of Twist1 in lung cancer tissues was significantly higher than in corresponding paracancerous tissues. Further data analysis using gene expression omnibus repository showed that Twist1 gene expression was slightly (approximately 1.1 fold) but significantly increased in tumor tissues from lung cancer patients compared to adjacent normal tissues. Moreover, we found that the overexpression of Twist1 was significantly associated with reduced survival of lung cancer patients, which was consistent with previous studies in primary NSCLC [32, 33]. However, different from these previous studies, we did not find that the overexpression of Twist1 was correlated with clinicopathological parameters. This discrepancy might be explained by the fact that the samples collected in the present study were from NSCLC tumors during surgical resection, among which most patients were at the clinical stage I-IIIA. On the other hand, a limited sample size has been used in the present study, and a future study using a larger sample from different histologic subtypes of lung cancer is needed to confirm the observed relationship between Twist1 and clinicopathological parameters. Although poor survival in NSCLC patients with Twist1 overexpression could be explained by its function as an important EMT regulator, this observation could also be related to other important molecular changes in NSCLC, such as epidermal growth factor receptor (EGFR), K-ras, and/or p53 mutations. In fact, the potential interactions between Twist1 and EGFR, K-ras, or p53, in cancers have been reported [15, 38–41]. For instance, Twist1 expression is associated with EGFR mutation in lung adenocarcinoma from non-smokers, and a cooperative effect between EGF pathway activation and Twist1 reactivation promotes EMT in EGFR mutated lung cancer [40]. Further studies are needed to elucidate the relationships between Twist1 and other molecular alterations in NSCLC and its importance for prognosis prediction.

The overexpression of Twist1 was associated with tumor invasion and metastasis, and inhibition of Twist1 with siRNAs suppressed mammary carcinoma cell metastasis [42]. The potential function of Twist1 was investigated by silencing in H1650 cells or overexpressing in H1975 cells. We have demonstrated that Twist1 has the oncogenic potential for proliferative growth in these two NSCLC cell lines. The silencing of Twist1 leads to the suppression of cell invasion and clone formation. On the other hand, the overexpression of Twist1 leads to the promotion of cell invasion and clone formation. Different from that observed in H1650 and H1650shTw, the size of the colonies did not show a significant difference between H1975Over and H1975, which might be due to the different characteristics between H1650 and H1975. For instance, a relatively slower growth rate could potentiate a smaller colony size. Finally, we have shown that Twist1 downregulation inhibits tumor growth in a nude mouse xenograft model, suggesting that the inhibition of Twist1 may be a potential therapeutic target. In addition to the well-documented effect of Twist1 on metastasis, recent work conducted by Morel *et al*. further highlighted the role of Twist1 in tumor initiation and primary tumor growth through the inhibition of key tumor suppressive mechanisms [43]. For instance, the synergistic effect of Twist1 and the EGF/RAS pathway promotes tumor initiation and development in breast and lung cancer models [40, 43]. Other EMT-inducing transcriptional factors, such as the SNAIL superfamily and ZEB family members, can also inhibit oncogene-induced senescence and cell apoptosis [44], which is worth additional investigation.

We examined the expressions of a series of proteins involved in AKT pathways by comparing the differences between H1650 and H1650shTw cells. We have first revealed that the downregulation of Twist1 decreased the protein expression of p-4E-BP1, but increased the gene expression of total 4E-BP1. These findings were further validated in H1975 and H1975Over cells. Additional data have indicated Twist1-correlated p-4E-BP1 overexpression in cancer tissues from NSCLC patients, among which, overexpressed p-4E-BP1 was associated with increased Twist1. These data suggest Twist1 involvement in the pathogenesis of lung cancer by regulating 4E-BP1 as well as p-4E-BP1. 4E-BP1 is a well-recognized eIF4E-binding protein that plays a critical role in protein synthesis and cell growth and survival [45, 46]. Translation is facilitated with the assistance of the eIF4F complex when eIF4E binds to the 5′-capped mRNA, which allows for the recruitment of associated scaffolding proteins [47]. 4E-BP1 by itself, but not the p-4E-BP1 form, binds to eIF4E and impedes formation of the initiation complex. As a consequence, translation is then blocked to favor apoptosis. A recent study has found that the phosphorylation site of 4E-BP1 is a possible prognosis predictor in patients with NSCLC, in which enhanced p-4E-BP1 Thr70 expression was highly correlated with low survival rates [30]. Moreover, a study conducted by Chang *et al*. found that long-term repeated, nose-only inhalation exposure of K-ras lung cancer model mice to 4E-BP1 significantly reduced tumor size, thereby providing exciting new possibilities in NSCLC treatment [48]. As observed in our study, the increased 4E-BP1 and decreased p-4E-BP1 after Twist1 knockdown could accelerate the binding affinity of 4E-BP1 to eIF4E and thereby result in the inhibition of cap-dependent translation. Although our present data did not provide enough support to show the interaction between Twist1 and 4E-BP1, our results, which showed that increased 4E-BP1 transcription and decreased p-4E-BP1 after Twist1 knockdown while decreased 4E-BP1 transcription and increased p-4E-BP1 after Twist1 overexpression, indicate that Twist1′s pathophysiological feature in lung cancer could act through 4E-BP1 regulation. Future studies using electrophoretic mobility shift assay and chip assay would provide additional evidence to determine the direct relationship between 4E-BP1 and Twist1.

Besides the changes observed for 4E-BP1 and p-4E-BP1, we have also demonstrated that p-mTOR was decreased after Twist1 knockdown. Among many reported 4E-BP1-associated kinases, such as cyclin-dependent kinase 1 [49], ataxia-telangiectasia mutated (ATM) [50], PI3K-AKT [51, 52], and ERK1/2 [53], mTOR is the main phosphorylation pathway of 4E-BP1 [21]. Therefore, the decrease of p-4E-BP1 could be the consequence of Twist1-mediated downregulation of p-mTOR. In line with our findings, a recent study has found that Twist1 siRNA induced suppression of mTOR activity, based on the decrease in phosphorylation of mTOR and mTOR effectors (S6K and S6) in H1299 cells [17]. Dysregulation of the mTOR pathway has been well recognized to play an important role in cancer development. For instance, Shin *et al*. recently found that the amalgamation of PTEN(−)/p-AKT(+)/p-mTOR(+) expression and poor overall survival were correlated in patients with Stage I lung adenocarcinoma [54]. Taken together, our data have provided further evidence of making Twist1 a potential therapeutic target through regulating mTOR and 4E-BP1.

RT-PCR array is a useful and powerful assay to screen the expression changes for genes of interest, but caution is needed regarding its sensitivity, accuracy, validity, and reproducibility. A total of 13 genes showed significant changes in H1650shTw compared to H1650. These findings provide additionally supportive evidence for understanding the significance of Twist1 in NSCLC. For instance, consistent with our findings, FOS has been shown to be downregulated after Twist depletion in gastric cancer cells [11, 55]. PTGS2, which is downregulated after Twist1 knockdown, is frequently increased and considered as a potential therapeutic target in NSCLC [56]. p53 gene expression showed a significant increase (2.7 fold) in H1650shTw compared to H1650 by qRT-PCR, and a marginally significant increase (1.9 fold) by RT-PCR array. Additionally, cell lines with p53 wild type (such as A549 and H1650) are likely to have a higher expression of Twist1 than those with p53 mutant/null (such as H1975 and H358). Thus, Twist1 overexpression might contribute to the inactivation of p53 functions, and a direct interaction between Twist1 and wild type p53, reported by a recent study, has partially supported this hypothesis [41]. Moreover, the decreased p-4E-BP1 expression after Twist1 knockdown in H1650 cells could be indirectly related to the increased p53 transcription level as observed in this study. 4E-BP1 is dephosphorylated by a phosphatase controlled by p53 [45, 57, 58]. Therefore, increased p53 could enhance the activity of the phosphatase, which might further dephosphorylate 4E-BP1. Collectively, a plethora of signal transduction pathways, besides the ones found in the present study, may be mediated by Twist1. It should be noted that the potential upstream and downstream regulators of Twist1 in different contexts of cancer cells could vary, which requires further investigation.

In summary, our data further validate the oncogenic potential of Twist1 in lung tumorigenesis through regulating 4E-BP1 and its corresponding pathways. Twist1 could represent an attractive therapeutic target for NSCLC. A better understanding of Twist1′s roles in lung cancer progression is likely to have important clinical implications for both prognostic prediction and therapeutic targeting.

## MATERIALS AND METHODS

### Patients and tissue specimens

A total of 75 NSCLC patients, 39 males and 36 females, were recruited for this study after obtaining informed consent, among which 72 patients had lung adenocarcinoma and 3 patients had mucoepidermoid carcinoma of the lung. The average patient age was 58.8 ± 11.9 (range of 20–84 years old). One hundred fifty samples, including 75 lung cancer tissues and 75 matched paracancerous tissues, were collected from patients who underwent resection of the primary tumor between July 2004 and September 2009 at the Jinling Hospital of Nanjing University, China. Both the cancer tissues and the paracancerous tissues were independently confirmed by two professional pathologists. Pathologic data were collected after a histopathological investigation. The depth of tumor invasion was assessed according to the Union for International Cancer Control (UICC) classification criteria [34]. The status of lymph node metastasis and differentiation grade were assessed according to the World Health Organization (WHO) classification criteria [35]. Clinical information of all subjects was obtained from medical records at the Jinling Hospital of Nanjing University. None of the patients received treatment prior to surgery. All specimens were handled anonymously according to ethical and legal standards. Written informed consent for study participation was obtained from all patients. The present study was approved by the Investigation and Ethics Committee of the Jinling Hospital of Nanjing University.

### Immunohistochemistry (IHC)

Tissue microarray (TMA) was prepared using the 150 samples. Immunohistochemical analysis was performed in TMA as previously described [36]. Briefly, incubation with the primary antibodies was conducted overnight at 4°C for Twist1 (1:100 dilution; Sigma, St. Louis, MO, USA) and p-4E-BP1 (Ser65, 1:400 dilution; Cell Signaling Technology, Danvers, MA, USA). Negative controls consisted of omitting the primary antibody, incubating the slides with phosphate buffered saline (PBS), and replacing the primary antibody with normal serum. The slides were analyzed by two certified pathologists who were blinded to the clinical information. The slides were scored according to staining intensity: 0, no visible reaction; 1, weak expression; 2, moderate expression; and 3, high expression. The immunohistochemical analyses were repeated in two separate TMA slides. For statistical analysis, samples were categorized into 2 groups: low expression cases *versus* overexpression cases. Samples scored ≥ 2 were considered overexpression, while those scored ≤ 1 were considered low expression.

### Cell lines and cell culture

Human lung cancer cell lines A549, H226, H358, H460, H522, H596, H1650, and H1975 were purchased from the American Type Culture Collection (ATCC). The cells were cultured in RPMI-1640 medium and supplemented with 10% fetal bovine serum (FBS) (Invitrogen Inc., Carlsbad, CA, USA), 50 U/mL penicillin, and 50 mg/mL streptomycin, at 37°C in a humidified incubator with 95% air and 5% CO_2_ by volume. Cells were sub-cultured or plated for subsequent experiments until they approached approximately 80% confluence.

### Twist1 knockdown and overexpression in lung cancer cells

Twist1 knockdown or overexpression were performed in H1650 or H1975 cells, named as H1650shTw or H1975Over, respectively, using human-specific pRetroSuper Twist1-shRNA (shTw) or pcDNA-Twist1 (Over) (gifted by Dr. Sara Piccinin at CRO National Cancer Institute, Aviano, Italy) by the Plasmid Transfection Reagent Kit (Santa Cruz Biotechnology, Santa Cruz, CA) following the manufacturer's protocol. Briefly, 2 × 10^5^ cells/well were seeded in a 6-well plate and allowed to grow to 70% confluency. The Twist1 or control plasmids were mixed with transfection reagents, overlaid on the cells for approximately 6 h, and transferred into 2× growth medium for approximately 20 h. After transfection, fresh medium was added and the cells were incubated for an additional 48 h. Thereafter, H1650shTw or H1975Over cells were selected by puromycin (4 μg/mL) or G418 (500 μg/mL), respectively. The knockdown and overexpression of Twist1 expression after transfection were verified by RT-PCR and Western blot.

### Quantitative real-time PCR (qRT-PCR)

RNA isolation and qRT-PCR were conducted as described in our previous studies [36, 37]. Briefly, total RNA was isolated by the RNeasy Plus Mini Kit (Qiagen, Valencia, CA) and quantified by a Nanodrop 1000 spectrophotometer (Thermo Scientific, Waltham, MA). A one-step RT-PCR kit with SYBR green was used for amplification of total RNA (50 ng) using a CFX96 Real-Time PCR Detection System (Bio-Rad, Hercules, CA, USA). Specificity of the PCR products was confirmed by melting curve analysis. The threshold cycle (Ct) value for each reaction was calculated. The primer sequences were listed in Table 1. Data were normalized to the Ct value of glyceraldehyde 3-phosphate dehydrogenase (GAPDH) from the same sample and the fold-changes in the expression of each gene were calculated using the ΔΔCt method. A non-template control was included in each experiment.

**Table 1:** Forward and reverse primers of genes used in qRT-PCR

Primer	Forward primer (5′to 3′)	Reverse primer (5′ to 3′)
GAPDH	GGTGGTCTCCTCTGACTTCAACA	GTTGCTGTAGCCAAATTCGTTGT
4E-BP1	CTATGACCGGAAATTCCTGATGG	CCCGCTTATCTTCTGGGCTA
BRCA1	GAGCTCGCTGAGACTTCCTG	ACTCCAGACAGATGGGACACT
CD44	CCCAGATGGAGAAAGCTCTG	GTTGTTTGCTGCACAGATGG
CTNNB1	TGCAGTTCGCCTTCACTATG	ACTAGTCGTGGAATGGCACC
MDM2	ATCAGCAGGAATCATCGGAC	GTGGCGTTTTCTTTGTCGTT
mTOR	CCAACAGTTCACCCTCAGGT	GCTGCCACTCTCCAAGTTTC
p53	GGCCCACTTCACCGTACTAA	GTGGTTTCAAGGCCAGATGT

### Western blot analysis

Cells were lysed in RIPA lysis buffer on ice. The lysates were sonicated and then centrifuged at 13,000 × g for 5 min at 4°C to collect the supernatant. Protein concentrations were measured using the Bio-Rad Bradford protein assay. A total of 30 μg of protein per sample was separated by 12% SDS-polyacrylamide gel electrophoresis (SDS-PAGE) and then transferred to polyvinylidene fluoride (PVDF) membranes. The immobilized proteins were then incubated in blocking buffer containing 3% nonfat dry milk in Tris buffered saline (TBS) and 0.1% Tween 20 (1× TBST). After blocking, the membranes were probed with the primary antibody overnight at 4°C. The membranes were then incubated with horseradish peroxidase (HRP)-conjugated secondary antibody and chemiluminescence. The signals on membranes were captured by X-ray films. Relative densitometric digital analysis of protein bands was determined using Image J and normalized by the intensity of the housekeeping gene, α-Tubulin (Abcam, Cambridge, MA, USA).

### Wound healing assay

The wound healing assay (scratch assay) was performed to examine the migration ability of cells. Briefly, H1650, H1650shTw, H1975, and H1975Over cells were grown to full confluency in 6-well plates and incubated overnight in a serum-free starvation medium. The cell monolayer was scratched with a sterile, fine pipette tip and washed with fresh medium to remove detached cells from the plates. After cells were kept in an incubator in a serum-free culture medium for 48 h, the medium was replaced with PBS and the wound gap was observed, photographed, and measured under a Nikon microscope.

### Anchorage-independent growth assay

Anchorage-independent growth was determined by colony formation assay in soft agar following a previous protocol [36]. Briefly, cells were suspended in the RPMI medium containing 10% FBS and 0.35% agar, and 2 × 10^4^ cells were plated in a well of a 6-well culture plate with a layer of 0.7% solidified agar. The cells were fed every three days by adding 200 μL of RPMI/10% FBS. Colonies were stained using 0.005% crystal violet, visualized, and scored by an image analyzer under a microscope.

### Array-based SYBR Green RT-PCR

Constitutive gene expression profiling related to Twist1 signal transduction was performed using the Human PrimePCR Cancer Tier 1 H96 (Bio-Rad) based on the manufacturer's instructions. This 96-well PCR array profiles the expression of 88 genes and includes the controls for human genomic DNA contamination, reverse transcription, positive PCR control, RNA quality assay (RQ1 and RQ2), and 3 housekeeping genes [TATA-box binding protein (TBP), GAPDH, and hypoxanthine phosphoribosyltransferase 1 (HPRT1)]. Briefly, 102 μL of diluted cDNA synthesis reaction was mixed with an experimental cocktail containing 1,020 μL of 2× SYBR green qPCR master mix and 918 μL Milli-Q water to form the PCR master mixture. An aliquot of 20 μL of the mixture was added to each well of the 96-well PCR array. RT-PCR was performed on CFX96 Real-Time PCR Detection System (Bio-Rad) under the following conditions: 2 min at 95°C (cycle 1), followed by 40 cycles of 5 s at 95°C, and 30 s at 60°C. The Ct value for each reaction was calculated and the relative gene expression for each gene was normalized by 3 housekeeping genes.

### Nude mouse xenograft experiments

Housing and care of the animals was approved by the Texas Tech University Institutional Animal Care and Use Committee in accordance with the NIH Guidelines for the Care and Use of Laboratory Animals. Tumor xenograft experiments were performed using H1650 and H1650shTw cells. A total of 5 × 10^5^ cells in 100 μL of PBS were inoculated subcutaneously into the flank of female athymic nude mice at 7–8 weeks of age (Charles River Laboratory, Wilmington, MA). Tumor size was assessed by external measurement of the length (L) and width (W) of the tumor using a digital caliper (Thermo Fisher Scientific). Tumor volume (TV, mm^3^) was calculated using the following equation: TV = (L × W^2^)/2.

### Statistical analysis

The SPSS 22.0 software was used to complete the data analysis. χ2-test and/or non-parametric tests were conducted to analyze the relationship between Twist1 or p-4E-BP1 expression and clinicopathological characteristics. The association between Twist1 and p-4E-BP1 immunointensity of the same specimens was analyzed using the Spearman rank correlation test. Kaplan-Meier curves were used for survival analysis, and log-rank was determined based on the differences. One-way ANOVA or *t*-test was used for the gene/protein expression, wound healing, and migration assays, as appropriate. RT-PCR array was analyzed using the software from Bio-Rad. All quantitative data are shown as mean ± SD. In each case, *P* < 0.05 was considered statistically significant.
